# Age and Haplotype Variations within FADS1 Interact and Associate with Alterations in Fatty Acid Composition in Human Male Cortical Brain Tissue

**DOI:** 10.1371/journal.pone.0042696

**Published:** 2012-08-10

**Authors:** Erika Freemantle, Aleksandra Lalovic, Naguib Mechawar, Gustavo Turecki

**Affiliations:** 1 Department of Genetics, McGill University, Montreal, Quebec, Canada; 2 Department of Psychiatry, McGill University, Montreal, Quebec, Canada; 3 McGill Group for Suicide Studies, Douglas Mental Health University Institute, Montreal, Quebec, Canada; Charité-Universitätsmedizin Berlin, Germany

## Abstract

Fatty acids (FA) play an integral role in brain function and alterations have been implicated in a variety of complex neurological disorders. Several recent genomic studies have highlighted genetic variability in the fatty acid desaturase (FADS1/2/3) gene cluster as an important contributor to FA alterations in serum lipids as well as measures of FA desaturase index estimated by ratios of relevant FAs. The contribution to alterations of FAs within the brain by local synthesis is still a matter of debate. Thus, the impact of genetic variants in FADS genes on gene expression and brain FA levels is an important avenue to investigate.

**Methods:**

Analyses were performed on brain tissue from prefrontal cortex Brodmann area 47 (BA47) of 61 male subjects of French Canadian ancestry ranging in age from young adulthood to middle age (18–58 years old), with the exception of one teenager (15 years old). Haplotype tagging SNPs were selected using the publicly available HapMap genotyping dataset in conjunction with Haploview. DNA sequencing was performed by the Sanger method and gene expression was measured by quantitative real-time PCR. FAs in brain tissue were analysed by gas chromatography. Variants in the FADS1 gene region were sequenced and analyzed for their influence on both FADS gene expression and FAs in brain tissue.

**Results:**

Our results suggest an association of the minor haplotype with alteration in estimated fatty acid desaturase activity. Analysis of the impact of DNA variants on expression and alternative transcripts of FADS1 and FADS2, however, showed no differences. Furthermore, there was a significant interaction between haplotype and age on certain brain FA levels.

**Discussion:**

This study suggests that genetic variability in the FADS genes cluster, previously shown to be implicated in alterations in peripheral FA levels, may also affect FA composition in brain tissue, but not likely by local synthesis.

## Introduction

**Figure 1:**
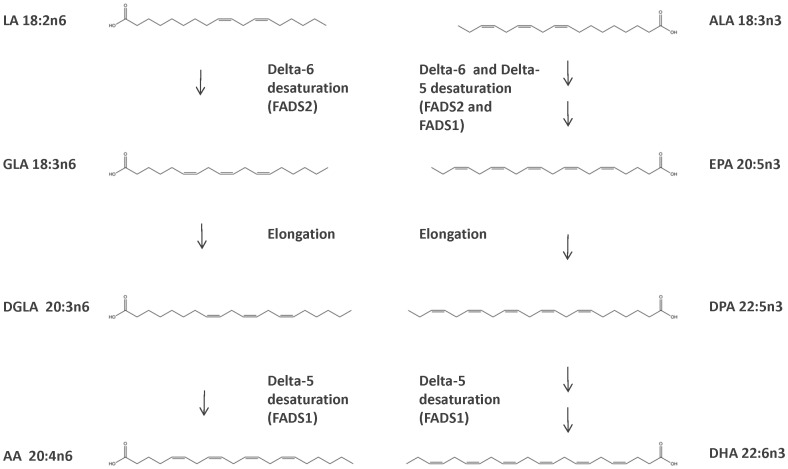
n6 and n3 LCPUFA biosynthesis pathway with relevant brain desaturation steps. Several FAs in the n3/n6 biosynthesis pathway are not reliably detectable in brain tissue.

To what extent the adult human brain relies on transport from peripheral circulation via receptor mediated uptake, passive diffusion, or local synthesis of the various n6 and n3 polyunsaturated fatty acids (FA) under physiological or pathological conditions is still a matter of some debate. Highly unsaturated FA, such as the major n6-FA arachidonic acid (AA) and major n3-FA docosahexanoic acid (DHA) are synthesised from their respective shorter chain FA linoleic acid (LA) and α-linolenic acid (ALA) through several precursors by a series of carbon chain elongation and desaturation steps, followed by a final β-oxidation step for DHA (summarised in Figure 1). LA and ALA are deemed essential FA as humans lack the desaturase enzyme to convert them from shorter chain FA. n6 and n3 FA account for approximately 25% of the lipid content of the brain. They are crucial in both structural roles in phospholipids in membranes and also as precursors for bioactive molecules, including eicosanoids and docosanoids, contributing to brain function. From both gene expression and FA desaturase activity studies represented as ratios of relevant FAs, altered brain FA has been implicated in a variety of cognitive, psychiatric, and neurodegenerative disorders such as bipolar disorder, schizophrenia, major depressive disorder (MDD), and Alzheimer’s disease, emphasizing the necessity to understand their regulatory mechanisms in the brain. [1]–[8] While there is not currently a consensus of which individual FA species are disadvantageous, it is typically thought that a reduced expression of FADS genes or a decrease in long chain desaturation products, especially DHA of the n3 FA class, is associated with an unfavorable outcome on cognition. [9].

Animal studies suggest preformed FA can readily cross the blood brain barrier either unesterfied from the plasma pool or via lipoprotein-mediated transport, and are the major source of animal brain n6 and n3 FA. [10], [11] In humans, genes responsible for chain elongation and desaturation of n6 and n3 unsaturated FA, such as FADS1 and FADS2, are expressed most highly in liver and adipose tissue but also in brain tissue, suggesting a role for FADS genes in desaturation of FA locally. FA composition in brain tissue is critical for proper brain function and has been suggested to impact events from neuroinflammation to neurotransmission. Whether fatty acid composition in the brain reflects peripheral composition or local synthesis has been investigated frequently in animals and seems to be largely under the control of an interaction between dietary intake, liver synthesis, and age, while the contribution of FA chain desaturation and elongation to FA levels within the adult human brain is more difficult to resolve. [12] However, in non-human primates it has been noted that while alterations in DHA intake, but not AA intake, result in changes in CNS composition of the respective FA suggesting the CNS may be differentially responsive to peripheral circulation. [13].

Several recent studies have highlighted the importance of genetic variation within the fatty acid desaturase (FADS 1/2/3) gene cluster as having an impact on unsaturated FA composition at peripheral locations, such as in serum, plasma, and erythrocyte membranes. [14]–[22] Sequence variation in FADS genes located on a 100 kb region of chromosome 11q12–13.1, particularly in FADS1 and 2, were shown to contribute to alterations in several FAs in the n6/n3 pathway and notably in the ratio of lipids representing their gene product activity. The use of ratios of FA as a proxy for desaturase activity index is commonly employed as direct measures of enzyme activity are not readily amenable. [22], [23] The FADS desaturase gene cluster includes a region in high linkage disequilibrium (D′>0.9) spanning a 48 kb region (Figure S1). [24], [25] Due to their similar chromosomal localisation and intron/exon patterns it has been suggested that the FADS genes arose from evolutionary duplication events with subsequent divergence in FA preference for desaturation activity. [26] Of note, FADS1 and 2 are located in a head to head orientation suggesting that variants located between the two upstream of the transcription start site would likely alter expression of both FADS1 and 2 by a variety of potential mechanisms including methylation differences or production of alternative transcripts (AT). Brenna et al. have recently characterised a number of AT for FADS2 in non-human primates and have shown that they are indeed expressed in many different tissues including the brain. [27]–[30] Several studies have since examined variants in association with inflammatory disorders, depression, and cardiovascular disorders, and general cognition underscoring their importance in human physiology. [31]–[34] Minor alleles associated with lower FADS1 activity and higher FADS2 activity largely result in higher DGLA and lower AA. AA is a precursor for inflammatory molecules and thus it is thought that minor variants may decrease risk of inflammatory disorders through mediating AA levels. It is thought that very little DHA is synthesised de novo, and most studies do not report an association in DHA by genotype supporting the importance of preformed DHA intake, [35] In a combined study of gene variants and FA intake, variations in FADS genes associated with lower FADS1 function was suggested to interact with FA intake with positive consequences on cognitive function. [36].

Determinants of FA levels in the periphery are likely a consequence of several interacting factors including intake and gene expression levels. As of yet, it is unknown whether genetic sequence variation in FADS genes contribute to alterations in gene expression of the desaturase enzymes in relation to alterations in FA levels in humans, or importantly, in human brain tissue. Integrated measures of genetic variant, gene expression, enzyme activity, and total FA levels are elemental to elucidate contributing factors to brain lipid levels. Thus, the objective of this study was to examine the relationship between genetic variation within FADS genes, the expression of FADS genes and, using relevant desaturase FA product/precursor ratios as a proxy of desaturation index, estimated FADS activity.

## Methods

### Post Mortem Human Brain Tissue

All subjects were males of French Canadian ancestry ranging in age from young adulthood to middle age (18–58 years old), with the exception of one teenager (15 years old).Brain tissue from Brodmann area 47 (BA47) was obtained from the Quebec Suicide Brain Bank (QSBB; www.douglas.qc.ca/suicide). Approval for this study was granted by the Douglas Hospital Institutional Review Board in accordance with the 1964 Declaration of Helsinki, and written informed consent was obtained from each participating family. Cause of death was ascertained by the Quebec Coroner’s Office in Montreal. The sample consisted of a mix of subjects who died by natural or accidental causes (N = 22, age μ±SD = 37±12) and subjects who died by suicide with (N = 16, age μ±SD = 34±11) or without a history of MDD (N = 23, age μ±SD = 35±10). All deaths were sudden and without prolonged agonal state, and none of the samples analyzed in this study came from individuals with a neuropathological illness. BA 47 was chosen for this study given importance of the prefrontal cortex in brain function and cognition, that LC-FA are found in high concentration in prefrontal cortex grey matter, and the previous associations of FADS variants with cognitive function. [4], [37].

### Haplotype Tagging SNP Selection, DNA Sequencing, and Restriction Enzyme Digest

**Figure 2:**
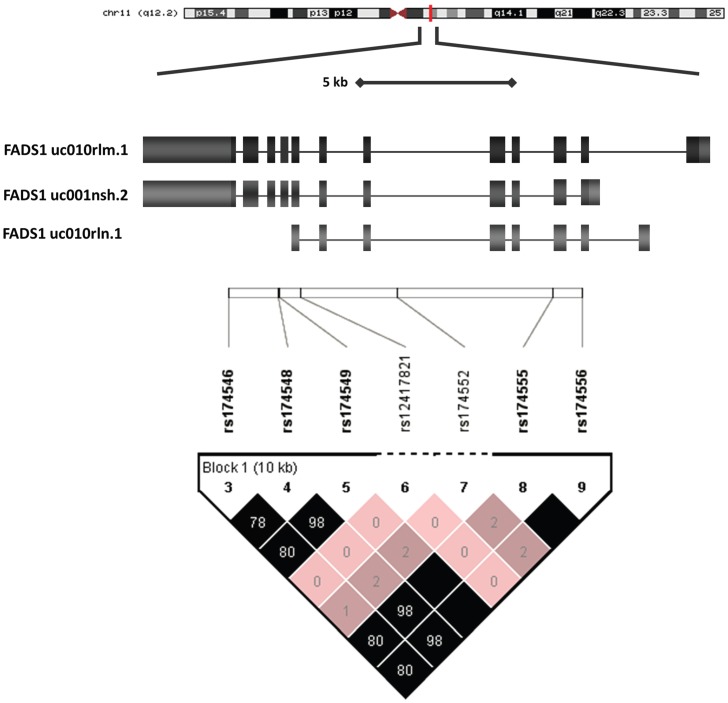
Gene region and haplotype tagging SNPs analysed in this study. Haplotype tagging SNPs were selected for FADS1 region to determine the impact of genetic variation on FADS expression and brain FA levels. This region is in high linkage with variants extending into the FADS2 promoter region.

Haplotype tagging SNP (htSNPs) were selected using publicly available HapMap genotyping data set from Utah residents with ancestry from Northern and Western Europe (CEU, version 3, release 2) in conjunction with Haploview (version 4.2). [10], [38] Criteria for SNP inclusion was a Hardy-Weinberg (HW) p-value cut-off of 0.001, minimum minor allele frequency of 0.05 and an r^2^ threshold of 0.8. [39] (Figure 2) Primers were designed to flank approximately 600 bp surrounding region of the selected htSNPs. DNA was extracted from blood or brain using the Qiagen DNeasy blood and tissue kit according to the manufacturer’s procedure. DNA integrity was estimated by nanodrop 260/280 and 260/230 ratios. Primers were designed using NCBI Primer-BLAST to flank an approximately 600 bp surrounding region of the selected htSNPs. Amplicons were purified and sequenced by the dideoxy chain termination Sanger sequencing method on Applied Biosystem’s 3730×l DNA Analyzer platform at the McGill University and Génome Québec Innovation Centre, quality of sequencing was determined by q-scores >50. Sequences were aligned using Bioedit (version 7.1.3.0) and Clustal W multiple alignment and verified by BLAST. Haplotype and SNP frequencies were assembled using Haploview (Tables 1 and 2). NCBI Genbank accession number series and primer details for each sequence set are available in Table 2. rs174555 amplicon was selected for validation by restriction digest with *BglI* (New England BioLabs), producing 3 distinct bands of 526 bp (T/T), 371+155 bp (C/T), 526+371+155 bp (C/C) (Figure S2). The restriction enzyme digest was in complete accord with the sequencing results.

**Table 1:** Haplotype characteristics.

Haplotype	CGT	GAC	CGC	p value
N	34	25	2	–
Age range (yr)	15–58	18–53	38–39	–
Age (yr)	35±12	36±10	39±1	0.83
Post-mortem interval	27±12	24±5.8	28±3.2	0.45
RNA integrity number	6.4±0.87	6.2±0.80	5.2±1.13	0.44
Brain tissue pH	6.5±0.3	6.5±0.3	6.7±0.2	0.52
**Frequency**	**0.664**	**0.27**	**0.06**	**–**

The haplotype frequencies were comparable to those specified in the CEU HapMap dataset. Subjects did not differ in terms of age, post-mortem interval, RNA integrity number, or brain tissue pH. Values represent mean ± standard deviation. Haplotypes reconstructed based on rs174548, rs174549, and rs174555. As only 2 subjects had the CGC haplotype, they were excluded from the subsequent analyses.

**Table 2:** Characteristics of SNPs analysed in this study.

SNP	HWp-value	Minor allele frequency	Alleles	GenBankaccession series	Primers 5′ → 3′
rs174546	0.201	0.369	C:T	JQ773028–JQ773088	F: TCACCAACTACCTGCATGTGCCA
					R: TGCTGTCAGCCTTCGCCGACAT
rs174548	0.167	0.27	C:G	JQ773089–JQ773142	F: TACCTGGGTGGAAACCCAGTCCA
					R: TGAAGGGGACATGCAAGAAGGGC
rs174549	0.167	0.27	G:A	JQ773089–JQ773142	F: TACCTGGGTGGAAACCCAGTCCA
					R: TGAAGGGGACATGCAAGAAGGGC
rs174555	0.118	0.336	T:C	JQ773200–JQ773260	F: TGGGCAATGTCAGTGGTGGGGA
					R: ACAGATGAGTTCCGGGAGCTGC

Minor allele frequencies were comparable to those specified in the CEU HapMap dataset and did not violate Hardy Weinberg equilibrium (HW p-value).

### Real-time PCR

RNA was extracted from brain using the Qiagen RNeasy lipid kit including DNase I incubation to remove contaminating genomic sequences. RNA quality was assessed by bioanalyser and RNA integrity number greater than 5 were deemed satisfactory. To quantify expression of refseq mRNA and to investigate presence of FADS AT expression, primers were designed using Primer3 to produce an approximately 200 bp amplicon for analysis by SYBR green quantitative real time PCR ABI 7900 Fast HT for different transcripts of FADS1 and FADS2 (summarised with primer details in Table 3). Relative quantitation was performed with expression normalised to GAPDH using a pool of all subjects as a calibrator. AT are designated by UCSC IDs. FADS1uc010rln.1 and FADS2uc010rlo.1 were outside the acceptable amplification cycle threshold for analysis and thus not included. FADS2uc001nsl.1 could not be resolved as a single AT and thus combined uc001nsl.1/uc001nsk.2 expression with comparison to separate expression of uc001nsk.2 was used to analyse these AT. A subset of subjects were also analysed by a commercially available Taqman assay for FADS1 using B-actin as an endogenous control to compare the effect of different chemistries and different endogenous controls on expression values. The correlation between the two measures was significant (r = 0.725, p = 0.00004, Figure S3). SDS software (version 2.4) was used for analysis of expression data.

**Table 3:** FADS transcription variants.

Gene	UCSC ID	Exons	Coding exons	Genomic size (bp)	UniProt ID	Protein size (aa)	Primers 5′ → 3′
FADS1	uc010rlm.1	12	12	17427	O60427	501	F: AGGAGCGGTGGCTAGTGAT
							R: GGCTGCTCTGGAGACAGTTC
FADS1	uc001nsh.2	11	11	13904	A8K0I7	360	F: ACTTGCAGGATCCCTTTGTG
							R: GAAGACATGGTTGGCCTTCA
FADS1	uc010rln.1	8	7	11448	A8K0I7	360	F: GGTCGAATGGGGACCTAGAG
							R: GGCTGCTCTGGAGACAGTTC
FADS2	uc001nsl.1	12	12	39113	O95864	444	F: CCGCAAGGTTTACAACATCA
							R: TCAGCAGGGGTTTCAAGAAC
FADS2	uc001nsk.2	10	10	35980	O95864-3	386	F: CACTGGTTTGTGTGGGTCAC
							R: GCTCACTGGTGCTCAATCTG
FADS2	uc010rlo.1	12	12	50950	B7Z634	413	F: GGTGAACTCGCTGATGTTGT
							R: CAGCAGGGGTTTCAAGAACT

Alternative transcripts analysed in this study.

### Fatty Acid Analysis

FAs from post-mortem human prefrontal cortex (BA47) were analysed as previously described. [40] Briefly, total lipids were extracted according to the Folch method [41], [42] in 2∶1 chloroform:methanol containing an internal standard and butylated hydroxyl toluene to prevent auto-oxidation during sample preparation. FAs were subjected to a hydrolysis and subsequent boron trifluoride-methanol methyl ester conversion. Fatty acid methyl esters were analysed by gas chromatographic separation-flame ionisation detection using helium as a carrier gas and identified by retention time of authenticated standards. FAs are expressed as weight percent of total FA. Fatty acid desaturase 1 (delta-5-desaturase, D5D) activity was estimated by proxy using the ratio of arachidonic acid (AA)/dihomo-gamma linolenic acid (DGLA), referred to as D5D index. Similarly, fatty acid desaturase 2 (delta-6-desaturase, D6D) activity, or D6D index, was estimated using the ratio of DGLA/linoleic acid (LA).

### Statistical Analyses

The variables analysed included relationships between measures of desaturase enzyme activity (AA/DGLA: D5D and DGLA/LA: D6D indices), quantities of n6 and n3 fatty acids (LA, DGLA, AA, ALA and DHA), gene expression of FADS1uc010rlm.1, FADS1uc001nsh.2, FADS2uc001nsl.1/uc001nsk.2, FADS2uc001nsk.2, and genetic variation in the FADS1 gene and the 2 reconstructed haplotypes (GCG haplotype was excluded as it only contained 2 subjects). Groups were not significantly different for post-mortem interval (PMI), RNA integrity number (RIN), or brain tissue pH (Table 1) and there was no significant correlation between PMI, RIN, pH and any measures analysed. Outliers were detected by box-plots and defined as greater than 2 standard deviations from the mean. Data was tested for normality by the Shapiro-Wilks test and non-normally distributed data was log10 transformed. One-way ANOVAs were performed and p-values were corrected by the number of measures for haplotype analysis (11), and the number of measures multiplied by the number of SNPs tested (44) for SNP analysis. Pearson or Spearman correlations were used depending on the normality of the data. Univariate general linear model including age as a covariate was used to determine effect sizes of haplotype and age interactions. SPSS was used for all statistical analyses (version 18). Graphpad Prism was used for graph preparations (version 5).

## Results

**Figure 3:**
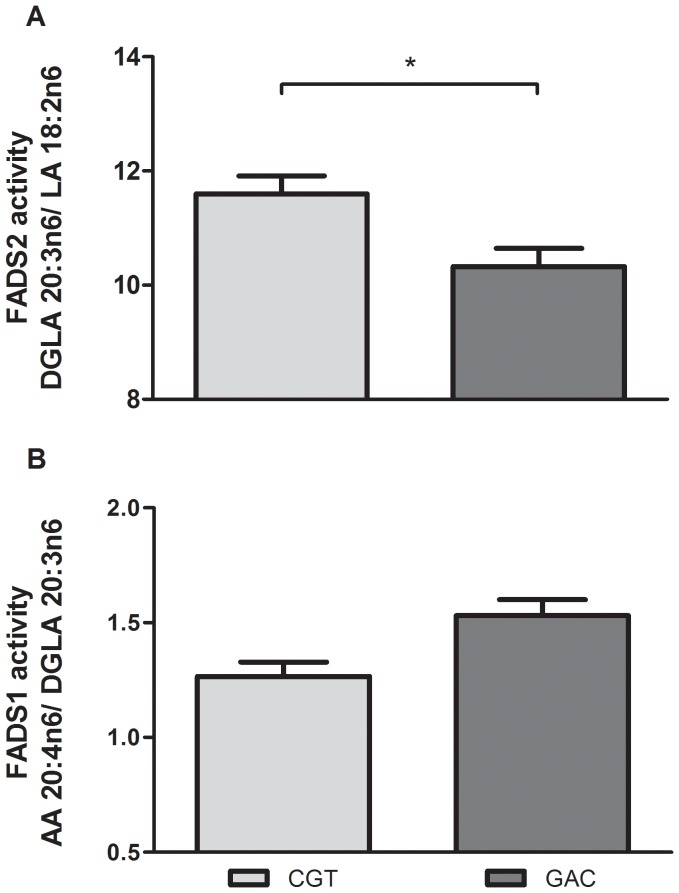
FA desaturation index by haplotype. Presence of minor haplotype is associated with alterations in estimated FADS2 and FADS1 activity (*p<0.05). (Sample size after outlier removal: FADS2 N = 34, 22; FADS 1 = N = 34, 24).

**Figure 4:**
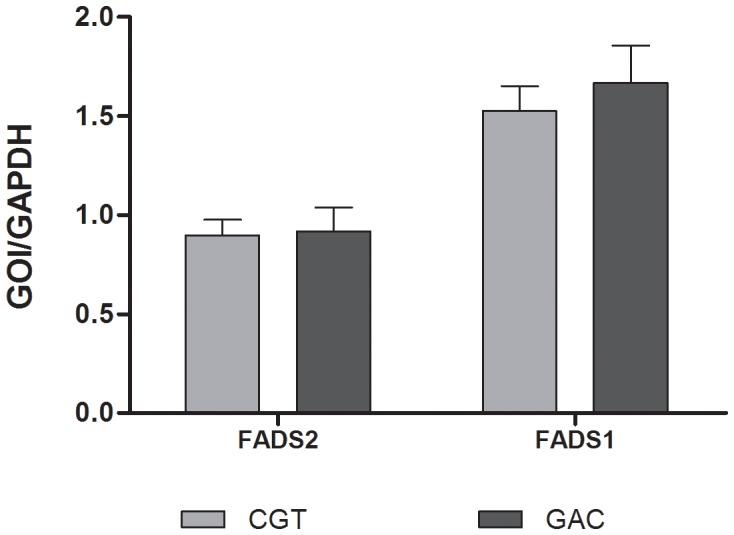
FADS2 and 1 gene expression by haplotype. Presence of minor haplotype is not associated with alterations in FADS2 and FADS1 gene expression.(Sample size after outlier removal: FADS2 N = 29, 20; FADS1 N = 31, 25).

**Figure 5:**
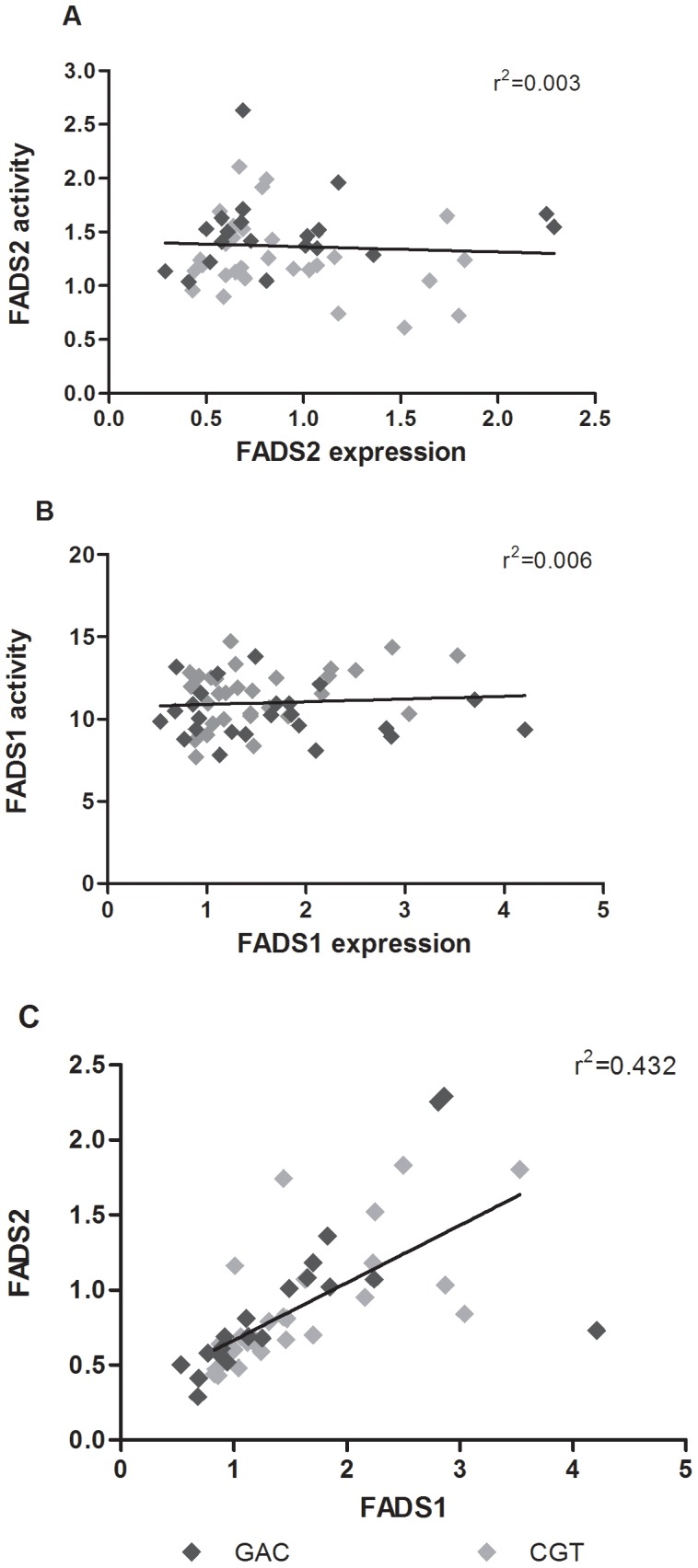
correlation of gene expression and desaturation activity with haplotype. FADS 1 and 2 activity is not correlated with expression nor is there an evident pattern of haplotype differences by scatter plot (Sample size after outlier removal: FADS2 N = 55; FADS1 N = 49). FADS1 and 2 gene expression are significantly correlated (N = 50,*p<0.05).

The haplotype and SNP frequencies were similar to those obtained from the HapMap CEU dataset and did not deviate significantly from Hardy-Weinberg equilibrium (Tables 1 and 2). Following correction for multiple testing, there was a significant effect of minor haplotype status on D6D index (p = 0.04, Figure 3A) and a trend for D5D index (p = 0.08, Figure 3B). There was no observed difference in gene expression for FADS1 or FADS2 according to haplotype (p = 0.530 and 0.874 for FADS1 and FADS2 respectively, Figure 4A and B), nor was there any correlation between gene expression and estimated activity (Spearman rho = 0.134 and 0.08 for FADS1 and FADS2 respectively); however, FADS1 and FADS2 gene expression were significantly correlated (r^2^ = 0.432, Spearman rho = 0.848 p = 7.5×10–15, Figure 5).

To rule out compensatory up-regulation in known alternative transcript expression between haplotypes, which has been recently implicated in FADS2 function in primates [27], [28] AT of FADS1 and FADS2 were also quantified and no differences were noted. Nevertheless, it is of interest that AT expression of both FADS1 and FADS2 were present in human brain tissue (data not shown).

**Figure 6:**
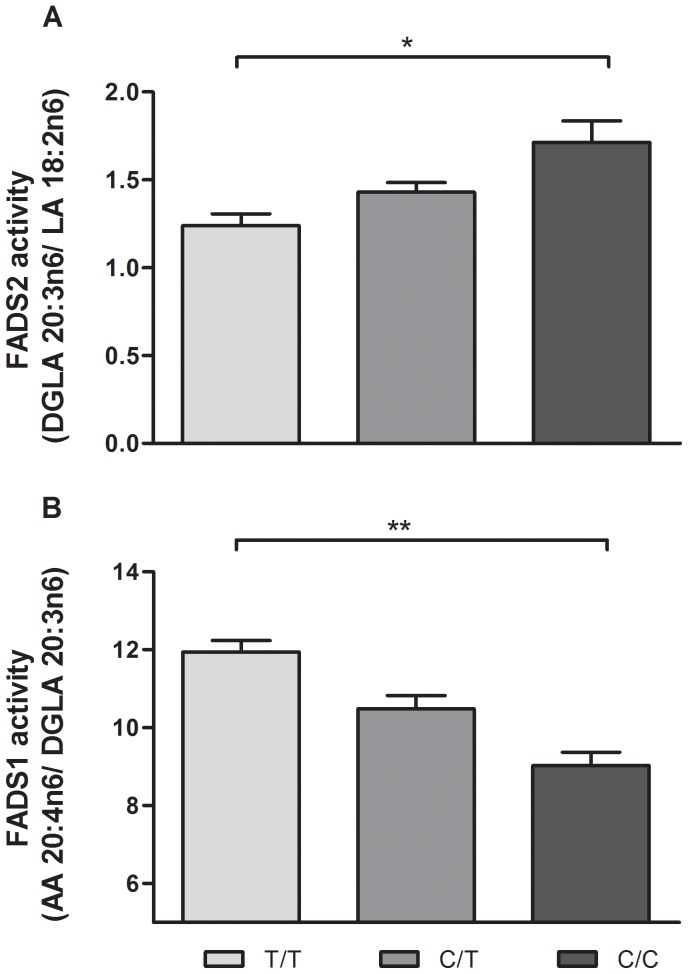
desaturation indices by rs174555 genotype. FADS desaturase activity shows an allele dosage dependent pattern. Following correction for multiple testing homozygous minor were significantly different compared to homozygous major (Sample size after outlier removal: FADS 2 activity N = 30, 21, 10; FADS 1 activity 30, 21, 9; *p<0.05).

**Figure 7:**
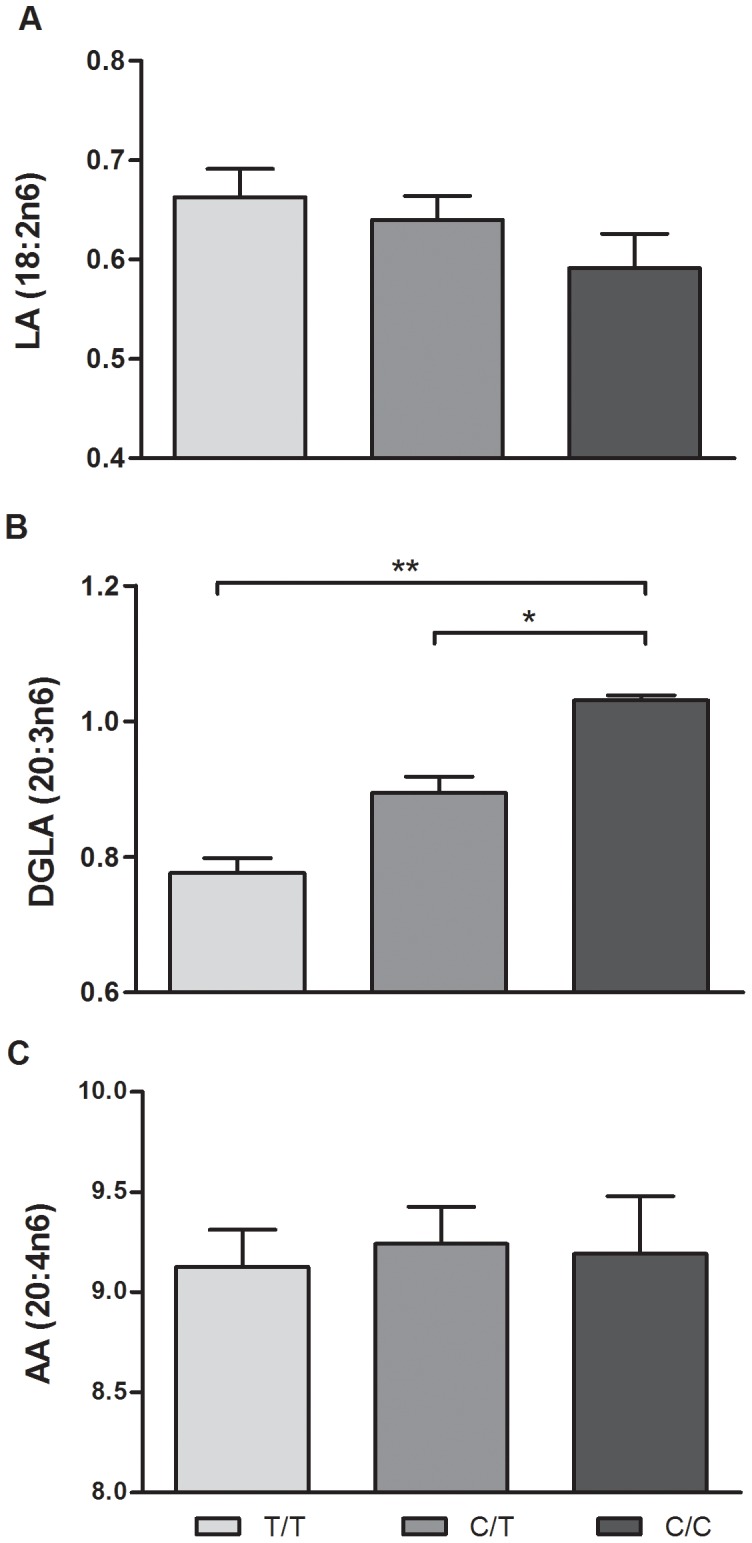
FA levels by rs174555. Following correction for multiple testing, homozygous minor were significantly associated with an increase in DGLA compared to homozygous major (*p<0.05) (Sample size after outlier removal: DGLA N = 30, 21, 7; LA, AA N = 30, 21,10).

For SNP analysis, as shown with rs174555, the alterations in D6D and D5D indices were observed in an allele dosage dependent manner, though after multiple testing correction, only homozygous genotypes were significantly different (T/T vs. C/C, p = 0.02 for D6D and p = 0.003 for D5D, Figure 6). All SNPs tested had a similar pattern for estimated desaturase index and gene expression (Figures S4 and S5). After correcting for multiple testing, no differences were observed for LA or AA, whereas minor variants associated with a significant increase in DGLA (p = 0.008, Figure 7).

**Figure 8:**
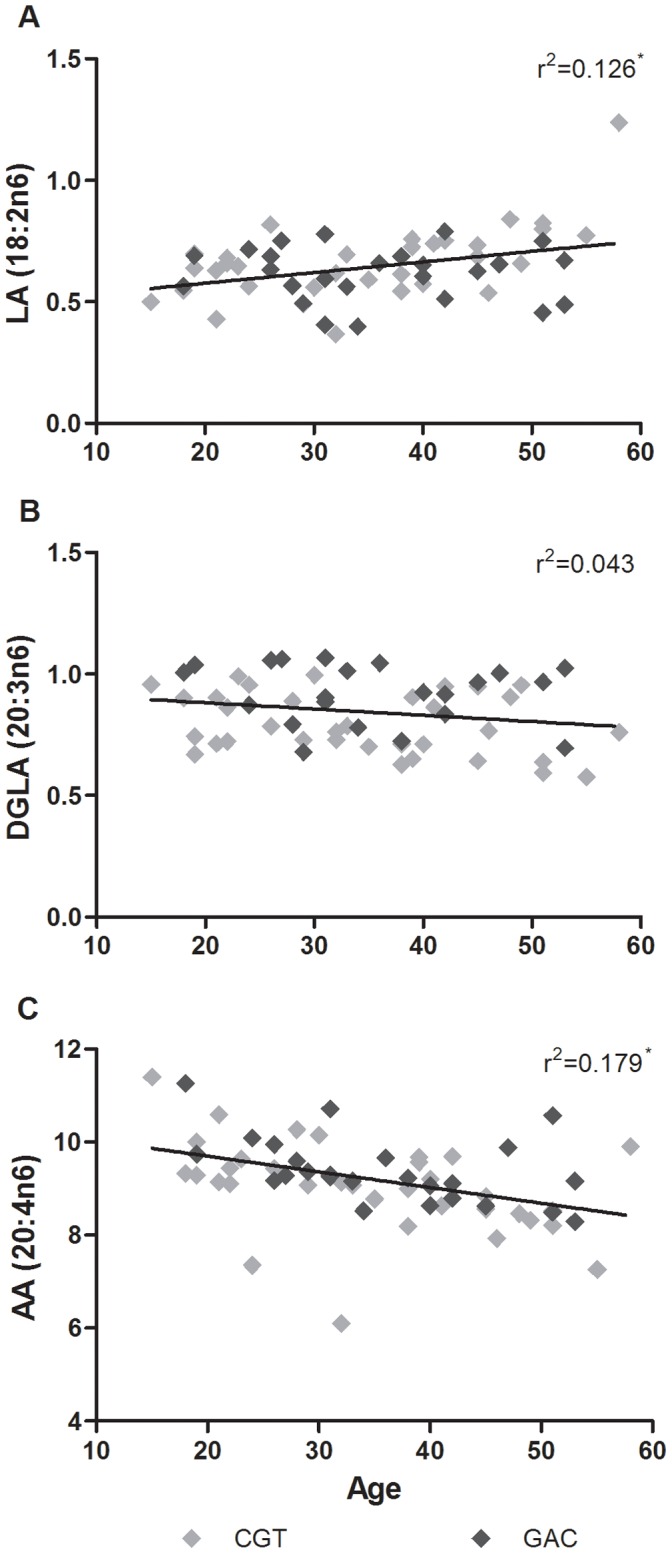
correlations of age and FA levels. LA and AA are significantly correlated with age (*p<0.05). General linear modelling was employed to determine the relative effect of age and haplotype status on lipid levels and desaturation indices (Sample size after outlier removal: LA N = 61; DGLA N = 58; AA N = 61).

There was no association of psychiatric diagnosis of subjects with or without MDD and haplotype (χ^2^ p value = 0.061) nor were there differences in analytical measures by psychiatric disorder (data not shown). Although, perhaps with increased sample size an association might be more readily detected. As age was found to be correlated with several measures including D6D (Pearson r = −0.353, p = 0.005), LA (Spearman rho = 0.293, p = 0.022) and AA (Spearman rho = −0.489, p = 0.00006, Figure 8) the interaction effect warranted follow-up. To investigate this further, univariate general linear model analysis was performed for all measures using age as a covariate. In the corrected model, there was a significant difference for D6D index (R^2^ = 0.320, p = 0.000021), D5D index (R^2^ = 0.121, p = 0.029), LA (R^2^ = 0.129, p = 0.021), DGLA (R^2^ = 0.251, p = 0.00047), and AA (R^2^ = 0.323, p = 0.001). No significant interaction effects were seen for age on n3 FA measures or FADS gene expression (data not shown).

## Discussion

The objective of this study was to investigate the relationship between FADS genetic variation, gene expression and estimated desaturase activity in post-mortem human prefrontal cortex. Though the genetic analysis was limited to SNPs in FADS1, this region of high linkage extends into the intergenic region between FADS1 and 2 located on chromosome 11; suggesting variation could potentially impact the promoter region of both genes. Thus, analysis was performed on FADS1 and 2 gene expression and desaturase activities as well. The impacts of different genetic variants on desaturase indices in brain tissue samples reported here are similar in direction and magnitude to alterations reported in the peripheral circulation. [22].

Minor haplotype within FADS1 was associated with an increase in the n6 FADS2 desaturation index (Figure 3A) and a decrease in FADS1 desaturation index (Figure 3B). Estimates of desaturase activity in the n3 pathway are not attainable in post mortem human brain tissue as the relevant FA are not reliably detectable. This may have obscured some potential associations; although no alterations were observed in absolute n3 FA levels.

While no other study has measured both FADS gene variation and expression in tandem with lipid profiles, a number of studies have investigated the relationship between two out of these three parameters. Kathiresan et al. assessed FADS1 gene variation and expression in 943 liver samples and found that FADS1 rs164547 T allele (major) was associated with increased expression of FADS1. [15] Furthermore, Lattka et al. investigated in cell cultures a common SNP (rs968567, Supplemental S1) upstream of the FADS2 transcription start site and found it to be associated with increased promoter activity due to differential binding activity of a transcription factor ELK1. This transcription factor was also shown to be significantly correlated with FADS2 in blood from a sample of 322 subjects. [43] Another recent study in cell culture suggested that expression of FADS1 AT may result in a differential expression of FADS2 which was associated with altered cellular localisation wherein FADS1 isoforms were localised to endoplasmic reticulum but excluded from the mitochondria. Finally, in cells stably transfected with both FADS1 AT and FADS2 the authors noted an accumulation of FA specifically of DGLA. [44] Taken together, these studies suggest that sequence variants do indeed contribute to differences in expression of FADS genes and it would be of importance to investigate AT expression in the periphery by variations in haplotype blocks.

Interestingly, in this study, there were no alterations in measures of gene expression for any of the FADS transcripts quantified, nor was there any observable relationship between desaturase gene expression and desaturase index. Only two previous studies have assessed the relationship between FADS gene expression and FA levels in human brain tissue. In one study, Liu et al. investigated the relationship between FADS2 expression and index of FADS2 activity (represented by AA/LA ratio) in the orbitofrontal cortex in a sample of 20 schizophrenics and 20 controls. While there was no relationship between FADS2 expression for either AA level, or for DGLA/LA ratio, these authors reported a significant correlation between expression and AA/LA ratio in controls. [8].

McNamara and colleagues also investigated the relationship between brain FA content and FADS1 and FADS2 expression during aging in the orbitofrontal cortex. In a sample of 30 subjects ranging from 29–80 years of age, they detected a positive correlation between FADS1 expression and AA/DGLA ratio, as well as between FADS2 and AA/LA ratio. While there was no relationship between age and FADS expression, there was a significant positive correlation between age and LA level, and a significant negative correlation between age and AA level. [7] It is worth noting that in both of these studies expression analysis was targeting all known variants of both FADS1 and 2. In agreement with McNamara et al, there was a significant positive correlation between age and brain LA levels and a significant negative correlation between age and AA levels in the present study (Figure 8). As such, analyses were undertaken to examine the relative contribution of genotype and age on brain FA. Haplotype accounted for more of the variance within DGLA levels, whereas both age and haplotype contributed to LA and AA levels.

The lack of difference in expression of FADS gene transcripts does not preclude small-scale fine-tuning of brain FA or transport of endogenously synthesised FA from other brain regions. However, the relatively large effect of genotype and the similarity to associations reported in human peripheral circulation suggest that the important variables contributing to prefrontal cortex brain FA levels might more largely depend on uptake from peripheral circulation, due to liver synthesis levels, with likely age-related differences in brain receptor-mediated uptake. Furthermore, it is not possible to dismiss a change in conversion of FA, especially AA, into downstream eicosanoids, such as prostaglandins, which are known to be biologically active inflammatory mediators and have important implications in brain function. Notably, expression of prostaglandin synthase genes have recently been identified to vary with age and psychiatric disorder in post-mortem human prefrontal cortex and this could be of interest for follow up investigations. [45].

A limitation of this study is the subject sample is composed only of males, thus no potential gender differences could be ascertained. Also, the fatty acids analysed were from total lipids and potential differences in lipid classes such as phospholipid profiles cannot be differentiated. An additional limitation is the necessity of extrapolation of metabolic states from post mortem tissue due to the lack of peripheral FA measures with the relatively small sample size for a SNP study. However, to date this is the largest sample size to combine gene sequence variants and gene expression measures in relation to brain FA levels. Of special importance, the group of Rapoport et al. have recently published a method using positron emission tomography to evaluate AA and DHA metabolism, suggesting the utility of employing this technique to examine how human brain AA/DHA signalling and metabolism are influenced by diet, aging, disease and genetics, which raises exciting possibilities to clarify many of the questions unresolved in relation to adult human brain FA function *in vivo*. [46].

In this study, the minor haplotype variant of the FADS1 gene was associated with an increase in DGLA level in human brain tissue. DGLA has been suggested to be an anti-inflammatory FA. [47] This is of relevance as the presence of minor haplotype status was found in one study to be protective against atopic eczema and allergic rhinitis. [14] Irrespective of AA levels, an increase in DGLA in brain tissue could potentially confer neuroprotective effects which would be of interest to investigate further.

### Conclusion

The mechanisms governing brain FA composition are vastly complex and in adult humans much remains to be resolved. This study suggests that genetic variability in the FADS genes cluster may also affect FA composition in brain tissue in a manner similar to changes previously shown in peripheral FA levels. This study also proposes that the lack of change in gene expression may reflect the dependence of uptake of preformed FA preferentially over local chain desaturation of precursor FA.

## Supporting Information

Figure S1 **Haplotype block structure of FADS1 and FADS2 gene region.** According to CEU HapMap dataset (with D’ values) spanning an 80 kb region including the intergenic region between FADS1 and 2.
(TIF)

Figure S2 **Example of** ***BglI*** **restriction enzyme digest.** Validation of rs174555 amplicon with *BglI* digestion produces distinct fragments of 526, 371, and 155 bp. Lane 1∶25 bp ladder lane 2 : C/C 371+155 bp lane 3 : C/T 526+371+155 bp lane 4 : T/T 526 bp.
(TIF)

Figure S3 **Correlation between FADS1 expression values normalised to GAPDH or β-actin.** Expression of FADS1 is comparable irrespective of endogenous control or chemistry used. β-actin was also evaluated using SYBR green chemistry but was determined to be less reliable than GAPDH. (N = 25, *p<0.05).
(TIF)

Figure S4 **Desaturase activity index by SNP genotype.** FADS desaturase activity is similar across SNPs tested. Following correction for multiple testing, rs174546 and rs174555 homozygous minor were significantly different compared to homozygous major (See table S1 for sample size after outlier removal,*p<0.05).
(TIF)

Figure S5 **FADS expression by SNP genotype.** FADS expression is similar across all SNPs tested. No differences were observed in expression levels (See table S1 for sample size after outlier removal).
(TIF)

Table S1 **Sample sizes after outlier removal by genotype for measures investigated in this study.**
(XLSX)

## References

[1] Conklin SM, Runyan CA, Leonard S, Reddy RD, Muldoon MF, et al (2010) Age-related changes of n-3 and n-6 polyunsaturated fatty acids in the anterior cingulate cortex of individuals with major depressive disorder. Prostaglandins Leukot Essent Fatty Acids 82: 111–119. 20060277 10.1016/j.plefa.2009.12.002 PMC2955405

[2] Conklin SM, Manuck SB, Yao JK, Flory JD, Hibbeln JR, et al (2007) High omega-6 and low omega-3 fatty acids are associated with depressive symptoms and neuroticism. Psychosom Med 69: 932–934. 17991818 10.1097/PSY.0b013e31815aaa42

[3] Conklin SM, Harris JI, Manuck SB, Yao JK, Hibbeln JR, et al (2007) Serum omega-3 fatty acids are associated with variation in mood, personality and behavior in hypercholesterolemic community volunteers. Psychiatry Res 152: 1–10. 17383013 10.1016/j.psychres.2006.10.006

[4] Muldoon MF, Ryan CM, Sheu L, Yao JK, Conklin SM, et al (2010) Serum phospholipid docosahexaenonic acid is associated with cognitive functioning during middle adulthood. J Nutr 140: 848–853. 20181791 10.3945/jn.109.119578 PMC2838625

[5] Fraser T, Tayler H, Love S (2010) Fatty acid composition of frontal, temporal and parietal neocortex in the normal human brain and in Alzheimer’s disease. Neurochem Res 35: 503–513. 19904605 10.1007/s11064-009-0087-5

[6] Milte CM, Sinn N, Howe PR (2009) Polyunsaturated fatty acid status in attention deficit hyperactivity disorder, depression, and Alzheimer’s disease: towards an omega-3 index for mental health? Nutr Rev 67: 573–590. 19785689 10.1111/j.1753-4887.2009.00229.x

[7] McNamara RK, Liu Y, Jandacek R, Rider T, Tso P (2008) The aging human orbitofrontal cortex: decreasing polyunsaturated fatty acid composition and associated increases in lipogenic gene expression and stearoyl-CoA desaturase activity. Prostaglandins Leukot Essent Fatty Acids 78: 293–304. 18499418 10.1016/j.plefa.2008.04.001 PMC2494852

[8] Liu Y, Jandacek R, Rider T, Tso P, McNamara RK (2009) Elevated delta-6 desaturase (FADS2) expression in the postmortem prefrontal cortex of schizophrenic patients: relationship with fatty acid composition. Schizophrenia research 109: 113–120. 19195843 10.1016/j.schres.2008.12.027 PMC8432756

[9] Lassek WD, Gaulin SJ (2011) Sex differences in the relationship of dietary Fatty acids to cognitive measures in american children. Front Evol Neurosci 3: 5. 22065957 10.3389/fnevo.2011.00005 PMC3206402

[10] Rao JS, Ertley RN, DeMar JC Jr, Rapoport SI, Bazinet RP, et al (2007) Dietary n−3 PUFA deprivation alters expression of enzymes of the arachidonic and docosahexaenoic acid cascades in rat frontal cortex. Mol Psychiatry 12: 151–157. 16983392 10.1038/sj.mp.4001887

[11] Spector AA (2001) Plasma free fatty acid and lipoproteins as sources of polyunsaturated fatty acid for the brain. J Mol Neurosci 16: 159–165; discussion 215–121. 10.1385/JMN:16:2-3:159 11478370

[12] Rapoport SI, Rao JS, Igarashi M (2007) Brain metabolism of nutritionally essential polyunsaturated fatty acids depends on both the diet and the liver. Prostaglandins Leukot Essent Fatty Acids 77: 251–261. 18060754 10.1016/j.plefa.2007.10.023 PMC2725409

[13] Hsieh AT, Brenna JT (2009) Dietary docosahexaenoic acid but not arachidonic acid influences central nervous system fatty acid status in baboon neonates. Prostaglandins Leukot Essent Fatty Acids 81: 105–110. 19524425 10.1016/j.plefa.2009.05.012

[14] Schaeffer L, Gohlke H, Muller M, Heid IM, Palmer LJ, et al (2006) Common genetic variants of the FADS1 FADS2 gene cluster and their reconstructed haplotypes are associated with the fatty acid composition in phospholipids. Hum Mol Genet 15: 1745–1756. 16670158 10.1093/hmg/ddl117

[15] Kathiresan S, Willer CJ, Peloso GM, Demissie S, Musunuru K, et al (2009) Common variants at 30 loci contribute to polygenic dyslipidemia. Nat Genet 41: 56–65. 19060906 10.1038/ng.291 PMC2881676

[16] Kwak JH, Paik JK, Kim OY, Jang Y, Lee SH, et al (2011) FADS gene polymorphisms in Koreans: association with omega6 polyunsaturated fatty acids in serum phospholipids, lipid peroxides, and coronary artery disease. Atherosclerosis 214: 94–100. 21040914 10.1016/j.atherosclerosis.2010.10.004

[17] Mathias RA, Vergara C, Gao L, Rafaels N, Hand T, et al (2010) FADS genetic variants and omega-6 polyunsaturated fatty acid metabolism in a homogeneous island population. J Lipid Res 51: 2766–2774. 20562440 10.1194/jlr.M008359 PMC2918459

[18] Lattka E, Rzehak P, Szabo E, Jakobik V, Weck M, et al (2011) Genetic variants in the FADS gene cluster are associated with arachidonic acid concentrations of human breast milk at 1.5 and 6 mo postpartum and influence the course of milk dodecanoic, tetracosenoic, and trans-9-octadecenoic acid concentrations over the duration of lactation. Am J Clin Nutr 93: 382–391. 21147856 10.3945/ajcn.110.004515

[19] Lattka E, Illig T, Koletzko B, Heinrich J (2010) Genetic variants of the FADS1 FADS2 gene cluster as related to essential fatty acid metabolism. Curr Opin Lipidol 21: 64–69. 19809313 10.1097/MOL.0b013e3283327ca8

[20] Koletzko B, Lattka E, Zeilinger S, Illig T, Steer C (2011) Genetic variants of the fatty acid desaturase gene cluster predict amounts of red blood cell docosahexaenoic and other polyunsaturated fatty acids in pregnant women: findings from the Avon Longitudinal Study of Parents and Children. Am J Clin Nutr 93: 211–219. 21106917 10.3945/ajcn.110.006189

[21] Tanaka T, Shen J, Abecasis GR, Kisialiou A, Ordovas JM, et al (2009) Genome-wide association study of plasma polyunsaturated fatty acids in the InCHIANTI Study. PLoS Genet 5: e1000338. 19148276 10.1371/journal.pgen.1000338 PMC2613033

[22] Bokor S, Dumont J, Spinneker A, Gonzalez-Gross M, Nova E, et al (2010) Single nucleotide polymorphisms in the FADS gene cluster are associated with delta-5 and delta-6 desaturase activities estimated by serum fatty acid ratios. J Lipid Res 51: 2325–2333. 20427696 10.1194/jlr.M006205 PMC2903808

[23] Warensjo E, Rosell M, Hellenius ML, Vessby B, De Faire U, et al (2009) Associations between estimated fatty acid desaturase activities in serum lipids and adipose tissue in humans: links to obesity and insulin resistance. Lipids Health Dis 8: 37. 19712485 10.1186/1476-511X-8-37 PMC2746208

[24] Marquardt A, Stohr H, White K, Weber BH (2000) cDNA cloning, genomic structure, and chromosomal localization of three members of the human fatty acid desaturase family. Genomics 66: 175–183. 10860662 10.1006/geno.2000.6196

[25] Stohr H, Marquardt A, White K, Weber BH (2000) cDNA cloning and genomic structure of a novel gene (C11orf9) localized to chromosome 11q12–>q13.1 which encodes a highly conserved, potential membrane-associated protein. Cytogenet Cell Genet 88: 211–216. 10828591 10.1159/000015552

[26] Nakamura MT, Nara TY (2002) Gene regulation of mammalian desaturases. Biochem Soc Trans 30: 1076–1079. 12440976 10.1042/bst0301076

[27] Park WJ, Reardon HT, Tyburczy C, Kothapalli KS, Brenna JT (2010) Alternative splicing generates a novel FADS2 alternative transcript in baboons. Mol Biol Rep 37: 2403–2406. 19693691 10.1007/s11033-009-9750-9 PMC3119850

[28] Brenna JT, Kothapalli KS, Park WJ (2010) Alternative transcripts of fatty acid desaturase (FADS) genes. Prostaglandins Leukot Essent Fatty Acids 82: 281–285. 20236814 10.1016/j.plefa.2010.02.011 PMC3045037

[29] Park WJ, Kothapalli KS, Reardon HT, Kim LY, Brenna JT (2009) Novel fatty acid desaturase 3 (FADS3) transcripts generated by alternative splicing. Gene 446: 28–34. 19573581 10.1016/j.gene.2009.06.016 PMC2740906

[30] Reardon HT, Park WJ, Zhang J, Lawrence P, Kothapalli KS, et al (2011) The polypyrimidine tract binding protein regulates desaturase alternative splicing and PUFA composition. J Lipid Res 52: 2279–2286. 21980057 10.1194/jlr.M019653 PMC3220295

[31] Standl M, Sausenthaler S, Lattka E, Koletzko S, Bauer CP, et al (2012) FADS gene cluster modulates the effect of breastfeeding on asthma. Results from the GINIplus and LISAplus studies. Allergy 67: 83–90. 21933193 10.1111/j.1398-9995.2011.02708.x

[32] Standl M, Sausenthaler S, Lattka E, Koletzko S, Bauer CP, et al (2011) FADS gene variants modulate the effect of dietary fatty acid intake on allergic diseases in children. Clin Exp Allergy 41: 1757–1766. 21793953 10.1111/j.1365-2222.2011.03833.x

[33] Xie L, Innis SM (2009) Association of fatty acid desaturase gene polymorphisms with blood lipid essential fatty acids and perinatal depression among Canadian women: a pilot study. J Nutrigenet Nutrigenomics 2: 243–250. 20395685 10.1159/000255636

[34] Lattka E, Illig T, Heinrich J, Koletzko B (2009) FADS gene cluster polymorphisms: important modulators of fatty acid levels and their impact on atopic diseases. J Nutrigenet Nutrigenomics 2: 119–128. 19776639 10.1159/000235559

[35] Glaser C, Lattka E, Rzehak P, Steer C, Koletzko B (2011) Genetic variation in polyunsaturated fatty acid metabolism and its potential relevance for human development and health. Matern Child Nutr 7 Suppl 227–40. 10.1111/j.1740-8709.2011.00319.x PMC6860603 21366865

[36] Morales E, Bustamante M, Gonzalez JR, Guxens M, Torrent M, et al (2011) Genetic variants of the FADS gene cluster and ELOVL gene family, colostrums LC-PUFA levels, breastfeeding, and child cognition. PLoS One 6: e17181. 21383846 10.1371/journal.pone.0017181 PMC3044172

[37] Diau GY, Hsieh AT, Sarkadi-Nagy EA, Wijendran V, Nathanielsz PW, et al (2005) The influence of long chain polyunsaturate supplementation on docosahexaenoic acid and arachidonic acid in baboon neonate central nervous system. BMC Med 3: 11. 15975147 10.1186/1741-7015-3-11 PMC1184078

[38] Gabriel SB, Schaffner SF, Nguyen H, Moore JM, Roy J, et al (2002) The structure of haplotype blocks in the human genome. Science 296: 2225–2229. 12029063 10.1126/science.1069424

[39] Haiman CA, Stram DO (2008) Utilizing HapMap and tagging SNPs. Methods Mol Med 141: 37–54. 18453083 10.1007/978-1-60327-148-6_3

[40] Lalovic A, Levy E, Canetti L, Sequeira A, Montoudis A, et al (2007) Fatty acid composition in postmortem brains of people who completed suicide. J Psychiatry Neurosci 32: 363–370. 17823652 PMC1963349

[41] Folch J, Ascoli I, Lees M, Meath JA, Le BN (1951) Preparation of lipide extracts from brain tissue. J Biol Chem 191: 833–841. 14861228

[42] Folch J, Lees M, Sloane Stanley GH (1957) A simple method for the isolation and purification of total lipides from animal tissues. J Biol Chem 226: 497–509. 13428781

[43] Lattka E, Eggers S, Moeller G, Heim K, Weber M, et al (2010) A common FADS2 promoter polymorphism increases promoter activity and facilitates binding of transcription factor ELK1. J Lipid Res 51: 182–191. 19546342 10.1194/jlr.M900289-JLR200 PMC2789778

[44] Park WJ, Kothapalli KS, Reardon HT, Lawrence P, Qian SB, et al.. (2012) A novel FADS1 isoform potentiates FADS2-mediated production of eicosanoid precursor fatty acids. J Lipid Res. 10.1194/jlr.M025312 PMC3540860 22619218

[45] Tang B, Capitao C, Dean B, Thomas EA (2012) Differential age- and disease-related effects on the expression of genes related to the arachidonic acid signaling pathway in schizophrenia. Psychiatry Res. 10.1016/j.psychres.2011.09.026 PMC3361581 22397921

[46] Basselin M, Ramadan E, Rapoport SI (2011) Imaging brain signal transduction and metabolism via arachidonic and docosahexaenoic acid in animals and humans. Brain Res Bull. 10.1016/j.brainresbull.2011.12.001 PMC3274571 22178644

[47] Umeda-Sawada R, Fujiwara Y, Ushiyama I, Sagawa S, Morimitsu Y, et al (2006) Distribution and metabolism of dihomo-gamma-linolenic acid (DGLA, 20:3n−6) by oral supplementation in rats. Biosci Biotechnol Biochem 70: 2121–2130. 16960355 10.1271/bbb.60057

