# Supplementary figures and images for: Age and Haplotype Variations within FADS1 Interact and Associate with Alterations in Fatty Acid Composition in Human Male Cortical Brain Tissue
Source: PLoS One. 2012 Aug 10;7(8):e42696. doi: 10.1371/journal.pone.0042696 (PMC3416866; doi:10.1371/journal.pone.0042696)

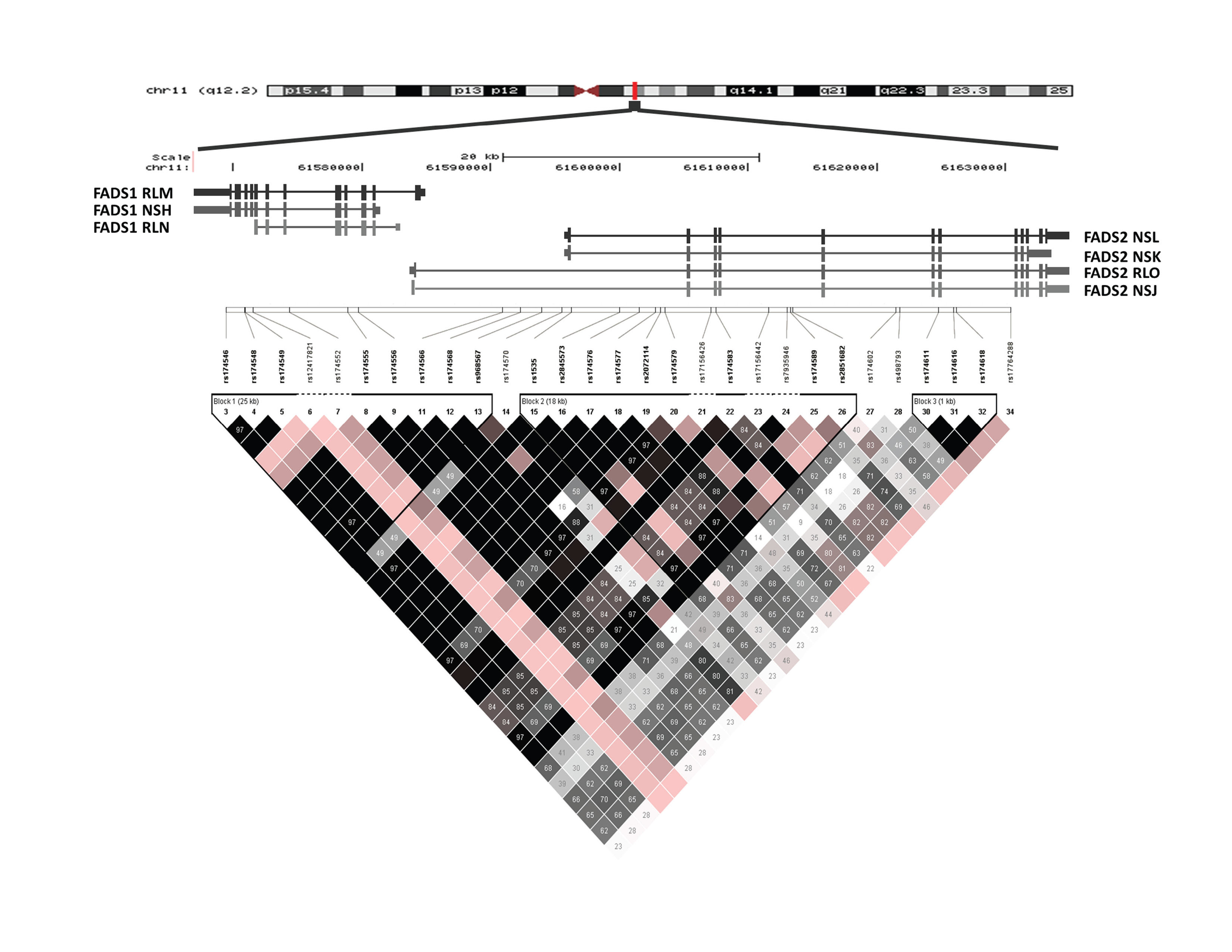

Supplement: Figure S1 — Haplotype block structure of FADS1 and FADS2 gene region. According to CEU HapMap dataset (with D’ values) spanning an 80 kb region including the intergenic region between FADS1 and 2. (TIF) [file pone.0042696.s001.tif]

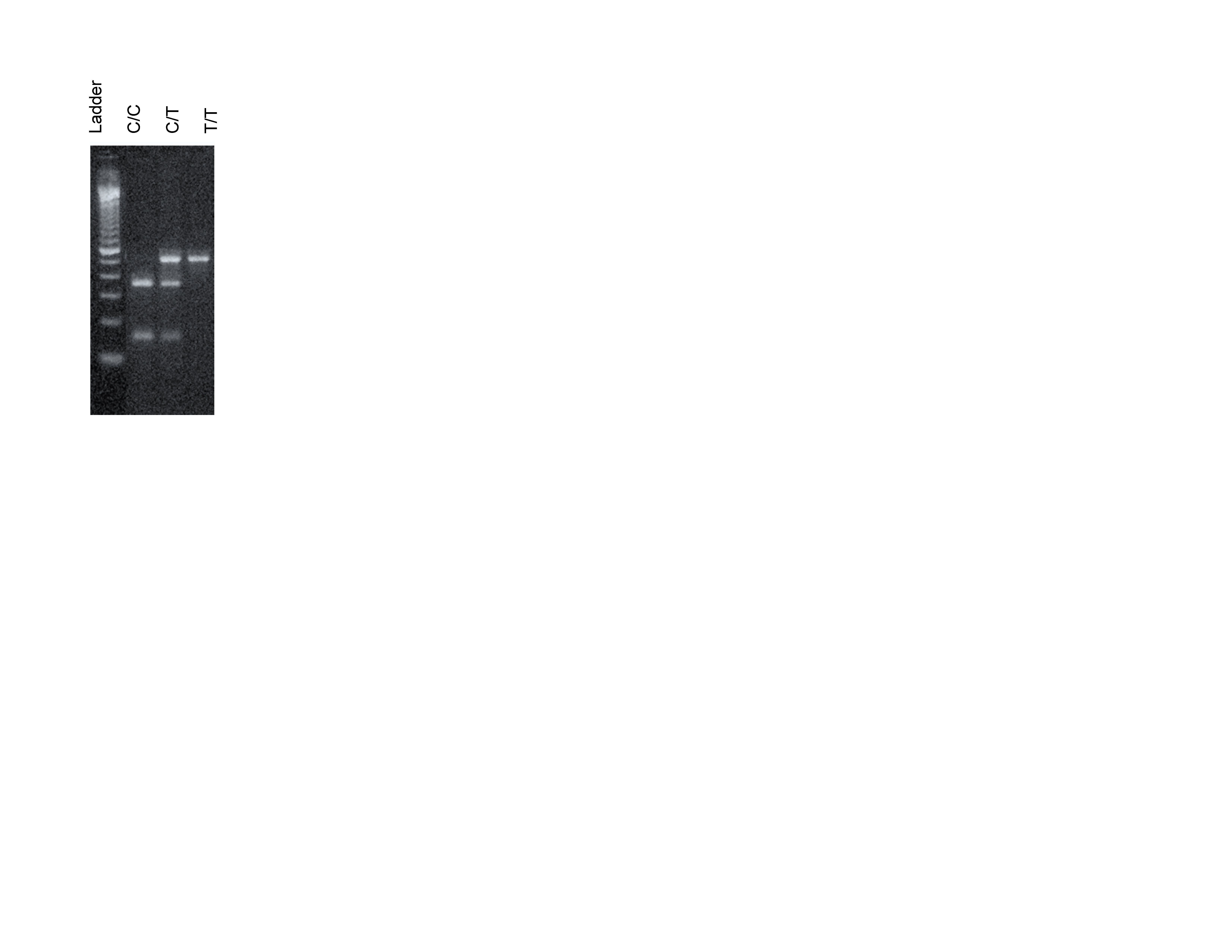

Supplement: Figure S2 — Example of BglI restriction enzyme digest. Validation of rs174555 amplicon with BglI digestion produces distinct fragments of 526, 371, and 155 bp. Lane 1∶25 bp ladder lane 2 : C/C 371+155 bp lane 3 : C/T 526+371+155 bp lane 4 : T/T 526 bp. (TIF) [file pone.0042696.s002.tif]

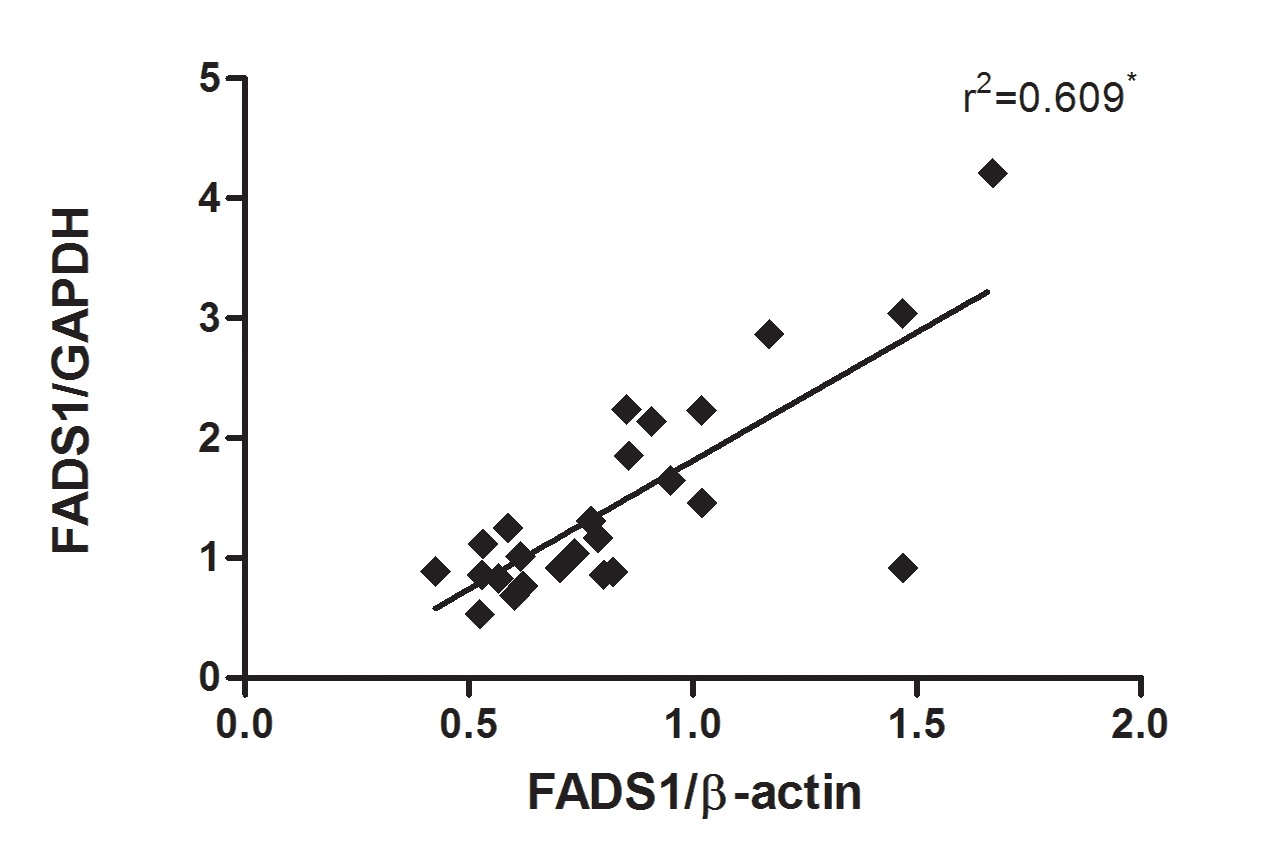

Supplement: Figure S3 — Correlation between FADS1 expression values normalised to GAPDH or β-actin. Expression of FADS1 is comparable irrespective of endogenous control or chemistry used. β-actin was also evaluated using SYBR green chemistry but was determined to be less reliable than GAPDH. (N = 25, *p<0.05). (TIF) [file pone.0042696.s003.tif]

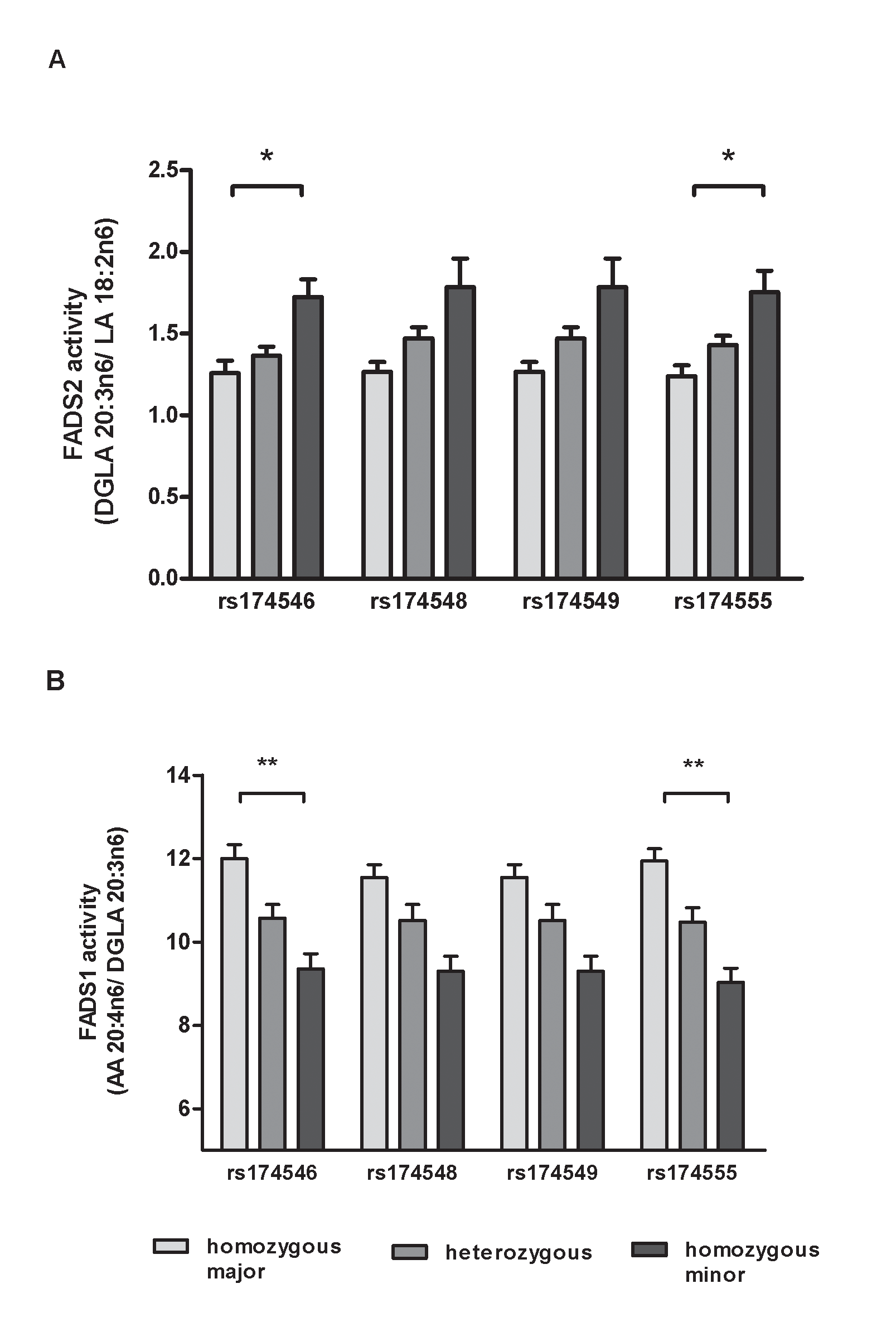

Supplement: Figure S4 — Desaturase activity index by SNP genotype. FADS desaturase activity is similar across SNPs tested. Following correction for multiple testing, rs174546 and rs174555 homozygous minor were significantly different compared to homozygous major (See table S1 for sample size after outlier removal,*p<0.05). (TIF) [file pone.0042696.s004.tif]

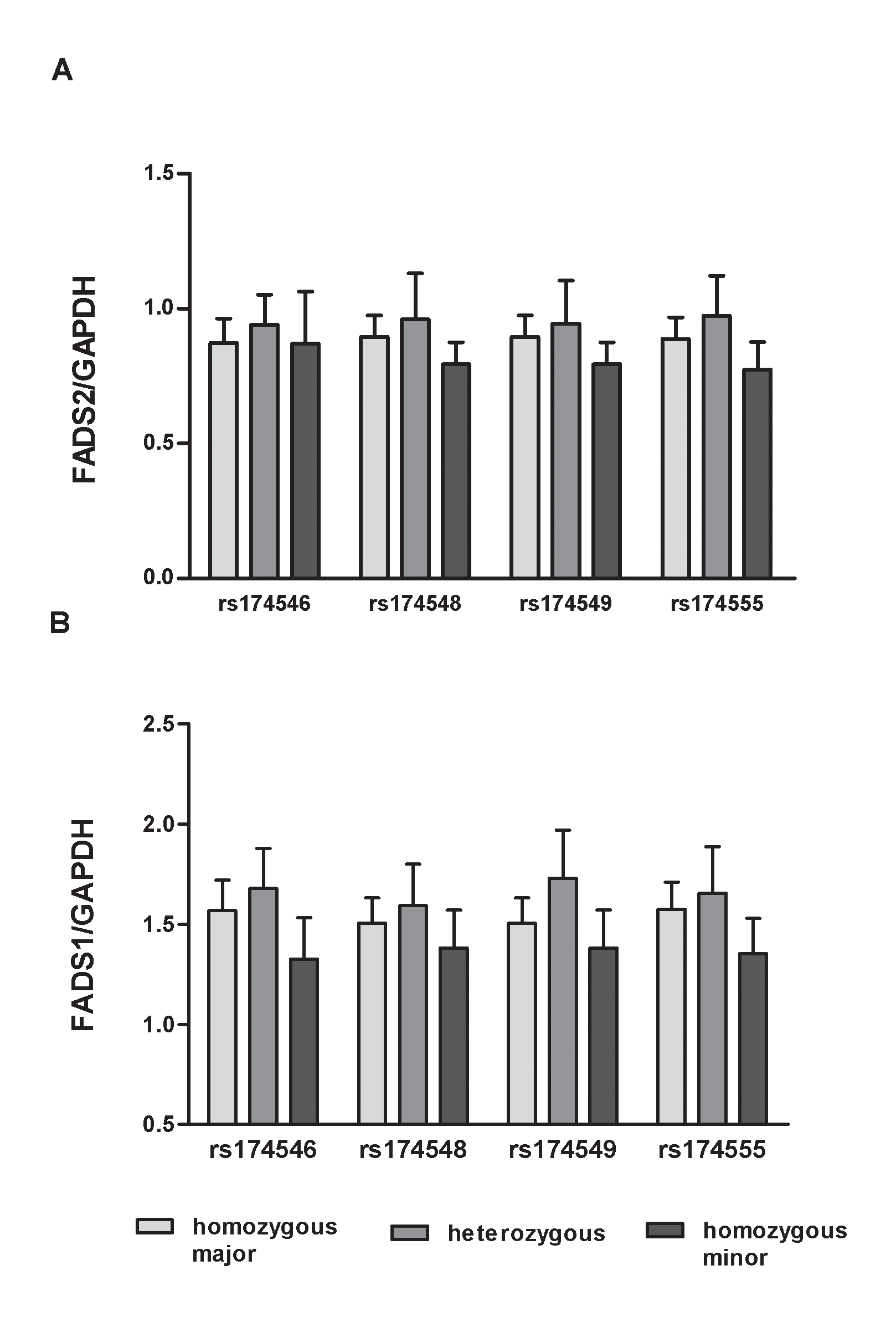

Supplement: Figure S5 — FADS expression by SNP genotype. FADS expression is similar across all SNPs tested. No differences were observed in expression levels (See table S1 for sample size after outlier removal). (TIF) [file pone.0042696.s005.tif]
